# Validation of the low anterior resection syndrome score in finnish patients: preliminary results on quality of life in different lars severity groups

**DOI:** 10.1177/1457496920930142

**Published:** 2020-06-18

**Authors:** Anu Carpelan, Eeva Elamo, Jukka Karvonen, Pirita Varpe, Sami Elamo, Tero Vahlberg, Juha Grönroos, Heikki Huhtinen

**Affiliations:** Department of Digestive Surgery, Turku University Hospital, Kiinamyllynkatu 4-8, Turku, 20520, Finland; Department of Digestive Surgery, Turku University Hospital, University of Turku, Turku, Finland; Department of Digestive Surgery, Turku University Hospital, University of Turku, Turku, Finland; Department of Digestive Surgery, Turku University Hospital, University of Turku, Turku, Finland; Department of Orthopaedics, Satakunta Central Hospital, Pori, Finland and University of Turku, Turku, Finland; Biostatistics, Department of Clinical Medicine, University of Turku, Turku, Finland; Department of Digestive Surgery, Turku University Hospital, University of Turku, Turku, Finland; Department of Digestive Surgery, Turku University Hospital, University of Turku, Turku, Finland

**Keywords:** Rectal neoplasms, adenocarcinoma, quality of life, surveys and questionnaires, translations, defecation, risk factors

## Abstract

**Background and Aims:**

Low anterior resection syndrome is common after anterior resection for rectal cancer. Its severity can be tested with the low anterior resection syndrome score. We have translated the low anterior resection syndrome score to Finnish, and the aim of this study is to validate the translation.

**Materials and Methods:**

The translated Finnish low anterior resection syndrome score and European Organisation for Research and Treatment of Cancer quality-of-life questionnaire-C30 and QLQ-CR29 questionnaires were sent to 159 surviving patients operated with anterior resection for rectal adenocarcinoma between 2007 and 2014 in a tertiary referral center. Psychometric properties of the translation were evaluated in comparison to quality-of-life scales and in different risk factor groups.

**Results:**

In the study, 104 (65%) patients returned the questionnaires. Of these, 56 (54%) had major low anterior resection syndrome, 26 (25%) had minor low anterior resection syndrome, and 22 (21%) had no low anterior resection syndrome. Patients with major low anterior resection syndrome had a significantly lower quality of life and more defecatory symptoms as assessed with the European Organisation for Research and Treatment of Cancer questionnaires compared with those with no low anterior resection syndrome. Patients operated with total mesorectal excision had significantly higher low anterior resection syndrome scores compared with those operated with partial mesorectal excision (median/interquartile range 32/15 and 29/11, respectively, *p* = 0.037). The test–retest validity of the translation was good with an intraclass correlation coefficient of 0.77 (95% confidence interval 0.51–0.90).

**Conclusions:**

The Finnish low anterior resection syndrome score is a valid test in the assessment of postoperative bowel function and its impact on the quality of life. It can be implemented to use during regular follow-up visits of Finnish-speaking rectal cancer patients.

## Introduction

Defecation disorders are frequent after anterior resection for rectal cancer. The usual combination of symptoms (urgency, fragmentation, incontinence) is called low anterior resection syndrome (LARS) and was described already in the 1990s^1^. The long-term prevalence of LARS after anterior resection has been reported to be up to 41%–56%^2–4^. Several risk factors for developing LARS have been described. Especially, total mesorectal excision (TME) instead of partial mesorectal excision (PME)^5–7^ and the use of preoperative long course chemoradiotherapy (CRT) or short course radiotherapy (RT)^5, 6, 8–10^ have been associated with major LARS. In some studies, younger age^6, 7^ and the formation or late closure of a protective ostomy^10, 11^ have also been significant risk factors for major LARS.

The impact of LARS on the quality of life of the patients was previously underestimated by surgeons^12^. To ease the assessment of the severity of defecatory symptoms, the LARS score was developed by Emmertsen and Laurberg^13^. It is a five-item questionnaire, by which patients can be divided into having no, minor, or major LARS. This division correlates with the amount of impact that bowel function disorders have on the quality of life of the patient. Since its development, LARS score has been widely adopted by the clinical and research communities and has been translated and validated in many languages^14–20^. In spite of growing awareness of the syndrome among colorectal surgeons, a recent study from the Netherlands still showed that postoperative bowel function is not routinely tested and more patient education is needed^21^.

To promote the evaluation and management of LARS in Finnish patients, we have translated the LARS score to Finnish. The purpose of this study is to validate the Finnish version of the LARS score, so that it can be also adopted for clinical use in Finland.

## Materials and Methods

Turku University Hospital is a tertiary referral center with centralized treatment of rectal cancer. All patients who underwent an anterior resection for rectal adenocarcinoma between 2007 and 2014 were collected from the hospital’s electronic patient records. Finnish-speaking patients who were alive and living without an ostomy at the moment of the study were included. Those with cognitive impairment (e.g. dementia or major psychiatric disease) or local recurrence of the carcinoma within the pelvis were excluded. Demographic and operative details of the included patients were collected from prospectively maintained electronic medical records.

The LARS score questionnaire was translated to Finnish from the previously validated English version^18^. Forward and backward translations were performed by colorectal surgeons, linguists, and native English speakers according to published protocols for translating health status questionnaires^22, 23^. Pilot testing of the translated questionnaire was done on 10 colorectal cancer patients at the outpatient clinic. The resulting Finnish LARS score questionnaire and scoring instructions used in this study are provided as Supplemental Appendices 1 and 2.

Eligible patients were contacted by mail in June 2018. They were sent an information leaflet, a patient informed consent-form, the Finnish LARS score questionnaire, the European Organisation for Research and Treatment of Cancer quality-of-life questionnaire (EORTC QLQ-C30; version 3.0)^24^ and EORTC QLQ-CR29 QLQs, and a prepaid return envelope. A second mailing was performed in August 2018 to increase the amount of participants. A subgroup of 23 patients received the LARS questionnaire twice in a 2-week interval to assess the test–retest reliability of the questionnaire.

Validity of the translation was tested according to previously published methodology^25^. A valid test gives similar results as other tests designed to measure the same construct (convergent validity). It also gives different results when measuring different constructs (discriminant validity). Convergent validity of the translation was tested by comparing the results of the Finnish LARS score with the results of previously validated Finnish versions of EORTC QLQs. Discriminant validity was evaluated by comparing the severity of LARS of patient groups with and without risk factors for developing LARS.

Data analysis was performed after omission of identifying information. The differences in the gender, operative details, or the use of CRT or RT between the groups of responding and non-responding patients were compared with chi-square or Fisher’s exact test. Two-sample *t*-test was used to test the difference in mean ages between the responding and non-responding patients. Intraclass correlation coefficient (ICC) was calculated to test the reliability of the Finnish LARS score in the test–retest group.

Global health status (QoL), functioning scales in the EORTC QLQ-C30 and symptomatic scales in QLQ-CR29 were compared between the LARS severity groups using Kruskal–Wallis test and further pairwise comparisons were done with Bonferroni-corrected Mann–Whitney U-test. Chi-square test was used to compare the categorical variables between the LARS severity groups. The difference in mean ages between the LARS severity groups was tested with one-way analysis of variance.

Values of *p* < 0.05 were considered statistically significant. Statistical analyses were done using IBM SPSS Statistics 25.0 for Windows (IBM Corp. Armonk, NY).

Research permission was obtained from the Institutional Review Board of Turku University Hospital. The study was approved by the ethics committee of the Hospital District of Southwest Finland.

## Results

Of 641 patients diagnosed with rectal adenocarcinoma in our unit during the study period, 159 were eligible; 104 (65%) of these patients returned the questionnaires. There were no significant differences in the age, gender, operative details or the use of CRT or RT between the groups of responding and non-responding patients. Background information about the responding group is presented in Table 1.

**Table 1.** Background information about the responding patients.

Variable	Responders (*n* = 104)
Age, years	72 ± 8
Gender (F/M)	40/64
Radiotherapy	
No radiotherapy	72 (69)
Short 5 × 5 Gy	26 (25)
Long 50.4 Gy with capecitabine	4 (4)
Postoperative radiotherapy	2 (2)
Type of operation	
TME	61 (59)
PME	43 (41)
Abdominal access	
Open	94 (90)
Laparoscopic	5 (5)
Laparoscopic converted to open	5 (5)
Protective ostomy	36 (35)
Time to closure of ostomy, months	7.6 (5.3)
Anastomotic leakage	11 (11)

F/M: female/male ratio; Gy: Gray; TME: total mesorectal excision; PME: partial mesorectal excision.

Values are given as mean ± SD, ratio, *n* (%) or median (interquartile range).

**Fig. 1.**
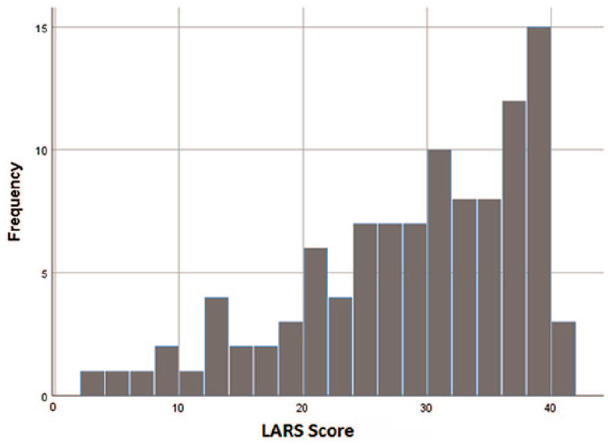
The distribution of LARS scores of the responding patients.

The questionnaires were filled at a mean time of 6.6 years (range 2.8–11.6 years, SD 2.4) after the anterior resection or closure of protective ostomy. The distribution of the LARS score is presented in Fig. 1. As a long-term functional result, 56 (54%) of the patients still had major LARS, 26 (25%) had minor LARS, and 22 (21%) had no LARS.

There was a clear, significant association of the LARS severity groups and quality of life, as measured with the EORTC QLQ-C30 and QLQ-CR29 instruments (Table 2). This indicates good convergent validity of the translation. When compared with patients with no LARS, those with major LARS had significantly lower scores (i.e. lower quality of life and lower level of functioning) on global health status/quality of life scale as well as on role, cognitive, and social functioning scales (Table 2). They also had higher scores (i.e. more symptoms) for symptomatic scales in CR29. The only significant difference between those with no LARS and those with minor LARS was in stool frequency. There was also no significant difference in the global quality of life of those with minor and those with major LARS, although the patients with major LARS had more flatulence and fecal incontinence as well as embarrassment of their bowel function (Table 2).

**Table 2.** Comparison of global health status/quality of life (QoL) and functional scales on EORTC QLQ-C30 and symptom scales relating to bowel functioning on EORTC QLQ-CR29 between different LARS severity groups.

Variable	No LARS (*n* = 22)	Minor LARS (*n* = 26)	Major LARS (n = 56)	*p* (all groups)
EORTC QLQ-C30				
Global health status/QoL (QL2)	80/83 (31)	76/75 (21)	67/67 (33)	0.012*
Physical functioning (PF2)	87/93 (17)	81/80 (22)	78/87 (38)	0.175
Role functioning (RF2)	92/100 (8)	87/92 (21)	82/83 (33)	**0.035** ^‡^
Emotional functioning (EF)	90/92 (17)	85/83 (29)	85/92 (25)	0.616
Cognitive functioning (CF)	94/100 (17)	88/83 (17)	84/83 (33)	**0.039** ^#^
Social functioning (SF)	97/100 (0)	91/100 (17)	84/100 (33)	**0.015** ^§^
EORTC QLQ-CR29				
Flatulence (FL)	32/33 (33)	33/33 (0)	51/33 (33)	**0.006** ^≠^
Fecal incontinence (FI)	4/0 (0)	16/0 (33)	31/33 (0)	**<0.001** ^†^
Sore skin (SS)	0/0 (0)	14/0 (33)	22/33 (33)	**<0.001** ^¶^
Stool frequency (SFr)	13/17 (17)	29/17 (17)	39/33 (33)	**0.001** ^×^
Embarrassment (EMB)	7/0 (0)	14/0 (33)	38/33(50)	**<0.001** ^$^

QoL: quality of life; LARS: low anterior resection syndrome; QLQ: quality of life questionnaires; PF: physical functioning; RF: role functioning; EF: emotional functioning; CF: cognitive functioning; SF: social functioning; FL: flatulence; FI: fecal incontinence; SS: sore skin; EMB: embarrassment.

Values are given as mean/median (interquartile range). *p*-values of <0.05 are considered statistically significant and bolded.

* Significant difference in pairwise comparison between no LARS versus major LARS (*p* = 0.018).

‡ Significant difference in pairwise comparison between no LARS versus major LARS (*p* = 0.033).

# Significant difference in pairwise comparison between no LARS versus major LARS (*p* = 0.039).

§ Significant difference in pairwise comparison between no LARS versus major LARS (*p* = 0.018).

≠ Significant differences in pairwise comparisons between no LARS versus major LARS (*p* = 0.030) and between minor LARS versus major LARS (*p* = 0.039).

† Significant differences in pairwise comparisons between no LARS versus major LARS (*p* < 0.001) and between minor LARS versus major LARS (*p* = 0.012).

¶ Significant difference in pairwise comparison between no LARS versus major LARS (*p* < 0.001).

× Significant differences in pairwise comparisons between no LARS versus major LARS (*p* < 0.001) and between no LARS versus minor LARS (*p* = 0.024).

$ Significant differences in pairwise comparisons between no LARS versus major LARS (*p* < 0.001) and between minor LARS versus major LARS (*p* = 0.003).

**Fig. 2.**
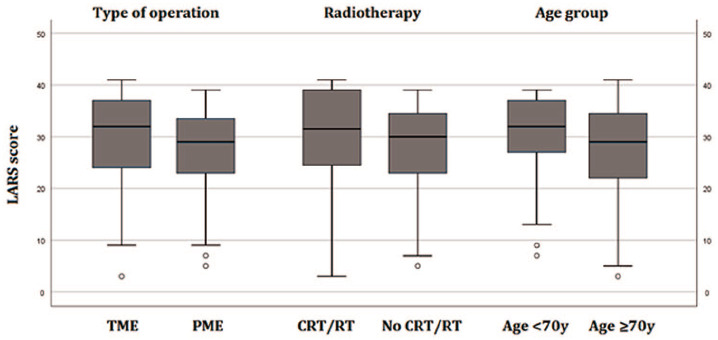
Distribution of the LARS score in different risk groups. A significant difference was found between TME and PME (*p* = 0.037), but not between radiotherapy (*p* = 0.132) or age groups (*p* = 0.080). TME: total mesorectal excision; PME: partial mesorectal excision; CRT: chemoradiotherapy; RT: radiotherapy.

Discriminant validity of the translation was assessed by comparing groups that were expected to differ in the severity of LARS. Analyses were performed for both the LARS score and LARS severity groups (no, minor and major LARS). There was a significant (*p* = 0.037) difference in the LARS score of patients operated with TME (median 32, interquartile range (IQR) 15) when compared with PME (median 29, IQR 11; Fig. 2). There was also a tendency to higher LARS scores for irradiated patients (*p* = 0.132) and younger patients (*p* = 0.080), but these differences were not significant.

**Fig. 3.**
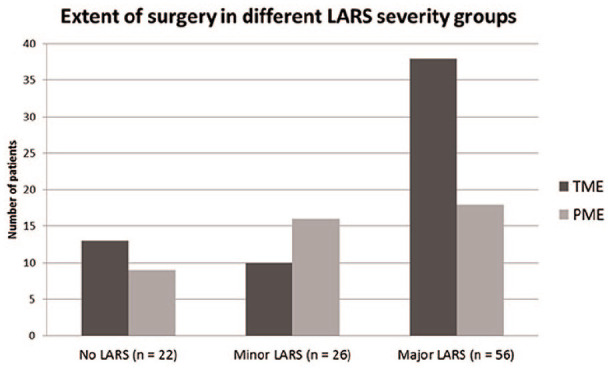
Number of patients operated with partial mesorectal excision (PME) and total mesorectal excision (TME) in the different LARS severity groups (*p* = 0.042).

When LARS severity groups were compared in relation to type of operation, CRT or RT and mean age, patients with major LARS were significantly more often operated with TME than patients with no or minor LARS (*p* = 0.042; Fig. 3). There was no significant difference in the mean age of patients with major, minor, or no LARS (71, 72, and 73 years, respectively). When analyzed separately for groups of patients under and over 70 years of age, 26 (63%) of the younger patients had major LARS compared with 30 (48%) of the older patients, but neither this difference was significant. The majority of the surviving patients were treated without radiotherapy (Table 1) but 37 (51%) of them still had major LARS. After CRT or RT the number of patients with major LARS was 19 (59%). This small difference was not significant.

In the test–retest group, 22 of the 23 patients returned the second LARS questionnaire; 17 (77%) of the patients were grouped to the same LARS severity category at both time points. Intraclass correlation coefficient (ICC) was 0.77 (95% CI 0.51–0.90) showing good reliability of the Finnish LARS score.

## Discussion

In this study, we have shown that the Finnish translation of LARS score has good psychometric properties. Convergent validity and test–retest reliability were excellent. When considering discriminant validity, the Finnish LARS score gave clearly differentiating results for patients with TME and PME operations, as expected. Patients operated with TME had significantly higher LARS scores than those operated with PME, which is in line with findings from previous studies^5–7^. LARS scores and LARS severity were also higher for patients treated with CRT or RT and in younger patients, but without statistical significances.

In studies reporting age to be a significant factor, the patients have been younger than in our study and the age limit has been set to 64 years^6, 7^. On the contrary, in two recent studies with mean ages of 63 and 70 years, age was not an independent risk factor^5, 9^. Altogether, of the seven published LARS score translation validation studies, only two have reported significant correlations between age and LARS score^14–20^. Thus the results of previous studies regarding age as a risk factor are inconsistent, although it would be logical for younger working-aged patients to experience more disturbances to their quality of life from LARS symptoms than for retired patients with more flexible time tables. As the mean age in our study was 72 years and only 20 patients were under 65 years old, it is possible to speculate that we did not have enough young patients to show a significant correlation.

As the included patients were long-term survivors of rectal carcinoma, the proportion of patients who had received CRT (4%) or RT (25%) in this study was unusually low (Table 1). During the same time frame, 51% of all patients with rectal adenocarcinoma treated in our hospital received CRT or RT^26^. This reflects the correct use of radiotherapy for patients with bad and ugly tumors only^27^, who unfortunately also have shorter overall survival or need an abdominoperineal excision instead of anterior resection. A follow-up study of the TME trial found a 56% prevalence of major LARS in irradiated patients 14 years postoperatively^3^. This percentage is in concert with the prevalence of major LARS after CRT or RT in our study. The reason for the surprisingly high proportion of major LARS in the group of patients treated without radiotherapy in our study is unclear. We can speculate that a patient who has major defecatory symptoms is more likely to fill in and return the questionnaires than a patient who is satisfied with his or her bowel movements.

Although the EORTC QLQs have previously been used to assess convergent validity of some of the LARS score translations^15, 18, 19^, pairwise comparisons of quality of life differences between all three LARS severity groups have only been performed in one multicenter study^28^. In this study, the authors presented significant differences between those with minor and major LARS, but the differences between patients with no LARS and minor LARS were small and clinically irrelevant although statistically significant. In our analysis, the EORTC QLQ-C30 could not pick up significant differences in the global quality of life between those with no and minor LARS or minor and major LARS. Nevertheless, there was a clear progression of defecatory symptoms when stepping up from no to minor LARS and from minor to major LARS, as assessed by the EORTC QLQ-CR29. Differences in the mean symptom scores of 10 or more are also considered clinically significant^29^.

The limitations of this study are its relatively small sample size and the fact that we needed to include patients from a long period of time to reach even this sample size. This may have caused bias to the LARS severity profile of the responding patient group. With a larger patient sample, the results on discriminant validity of the translation might have been stronger. But even with this sample size the directions of correlation between the Finnish LARS scores and risk factor groups were as anticipated. Thus we consider the discriminant validity of the translation to be satisfactory.

In conclusion, the translated Finnish LARS score is a valid test in the assessment of postoperative bowel function and its impact on the quality of life. It can be implemented to use during regular follow-up visits of Finnish speaking rectal cancer patients.

## Supplemental Material

sj-pdf-1-sjs-10.1177_1457496920930142 – Supplemental material for Validation of the low anterior resection syndrome score in finnish patients: preliminary results on quality of life in different lars severity groups Click here for additional data file. Supplemental material, sj-pdf-1-sjs-10.1177_1457496920930142 for Validation of the low anterior resection syndrome score in finnish patients: preliminary results on quality of life in different lars severity groups by Anu Carpelan, Eeva Elamo, Jukka Karvonen, Pirita Varpe, Sami Elamo, Tero Vahlberg, Juha Grönroos and Heikki Huhtinen in Scandinavian Journal of Surgery

sj-pdf-2-sjs-10.1177_1457496920930142 – Supplemental material for Validation of the low anterior resection syndrome score in finnish patients: preliminary results on quality of life in different lars severity groups Click here for additional data file. Supplemental material, sj-pdf-2-sjs-10.1177_1457496920930142 for Validation of the low anterior resection syndrome score in finnish patients: preliminary results on quality of life in different lars severity groups by Anu Carpelan, Eeva Elamo, Jukka Karvonen, Pirita Varpe, Sami Elamo, Tero Vahlberg, Juha Grönroos and Heikki Huhtinen in Scandinavian Journal of Surgery
