# Supplementary material for: Validation of the low anterior resection syndrome score in finnish patients: preliminary results on quality of life in different lars severity groups
Source: Scand J Surg. 2020 Jun 18;110(3):414–9. doi: 10.1177/1457496920930142 (PMC8551436; doi:10.1177/1457496920930142)
Supplement: sj-pdf-1-sjs-10.1177_1457496920930142 – Supplemental material for Validation of the low anterior resection syndrome score in finnish patients: preliminary results on quality of life in different lars severity groups [file sj-pdf-1-sjs-10.1177_1457496920930142.pdf]

Low Anterior Resection Syndrome Score – LARS Score / suomennos

Kysely suolen toiminnasta

Tämän kyselyn tavoite on arvioida suolen toimintaa. Rastita kunkin kysymyksen kohdalta vain yksi vastaus. Voi olla vaikeaa valita vain yksi, sillä oireet saattavat vaihdella päivästä toiseen. Valitse kuitenkin se vastaus, joka parhaiten kuvaa jokapäiväistä elämääsi. Jos sinulla on hiljattain ollut suolen toimintaan vaikuttanut tulehdus, älä ota sitä huomioon. Keskity vastaamaan kysymyksiin sen mukaan, miten suoli yleensä toimii.

Onko sinulla koskaan tilanteita, jolloin et pysty pidättämään ilmaa?

◻ Ei koskaan
◻ Kyllä, harvemmin kuin kerran viikossa
◻ Kyllä, ainakin kerran viikossa

Karkaako sinulta koskaan nestemäistä ulostetta?

◻ Ei koskaan
◻ Kyllä, harvemmin kuin kerran viikossa
◻ Kyllä, ainakin kerran viikossa

Kuinka usein ulostat?

◻ Yli 7 kertaa vuorokaudessa
◻ 4–7 kertaa vuorokaudessa
◻ 1–3 kertaa vuorokaudessa
◻ Harvemmin kuin kerran vuorokaudessa

Täytyykö sinun koskaan ulostaa uudelleen tunnin kuluessa edellisestä ulostuskerrasta?

◻ Ei koskaan
◻ Kyllä, harvemmin kuin kerran viikossa
◻ Kyllä, ainakin kerran viikossa

Onko sinulla koskaan niin voimakasta ulostustarvetta, että täytyy kiirehtiä vessaan?

◻ Ei koskaan
◻ Kyllä, harvemmin kuin kerran viikossa
◻ Kyllä, ainakin kerran viikossa
