# Supplementary material for: Validation of the low anterior resection syndrome score in finnish patients: preliminary results on quality of life in different lars severity groups
Source: Scand J Surg. 2020 Jun 18;110(3):414–9. doi: 10.1177/1457496920930142 (PMC8551436; doi:10.1177/1457496920930142)
Supplement: sj-pdf-2-sjs-10.1177_1457496920930142 – Supplemental material for Validation of the low anterior resection syndrome score in finnish patients: preliminary results on quality of life in different lars severity groups [file sj-pdf-2-sjs-10.1177_1457496920930142.pdf]

Low Anterior Resection Syndrome Score – LARS Score / Pisteytysohjeet

Kokonaispistemäärän saat laskemalla yhteen 5 kysymyksestä saadut pisteet.

Onko sinulla koskaan tilanteita, jolloin et pysty pidättämään ilmaa?

□ Ei koskaan	0
□ Kyllä, harvemmin kuin kerran viikossa	4
□ Kyllä, ainakin kerran viikossa	7

Karkaako sinulta koskaan nestemäistä ulostetta?

□ Ei koskaan	0
□ Kyllä, harvemmin kuin kerran viikossa	3
□ Kyllä, ainakin kerran viikossa	3

Kuinka usein ulostat?

□ Yli 7 kertaa vuorokaudessa	4
□ 4–7 kertaa vuorokaudessa	2
□ 1–3 kertaa vuorokaudessa	0
□ Harvemmin kuin kerran vuorokaudessa	5

Täytyykö sinun koskaan ulostaa uudelleen tunnin kuluessa edellisestä ulostuskerrasta?

□ Ei koskaan	0
□ Kyllä, harvemmin kuin kerran viikossa	9
□ Kyllä, ainakin kerran viikossa	11

Onko sinulla koskaan niin voimakasta ulostustarvetta, että täytyy kiirehtiä vessaan?

□ Ei koskaan	0
□ Kyllä, harvemmin kuin kerran viikossa	11
□ Kyllä, ainakin kerran viikossa	16

Kokonaispistemäärä: ________

Tulkinta:

0-20: Ei LARSia
21-29: Lievä LARS
30-42: Vaikea LARS
